# Effects of Submerged Macrophytes on Demography and Filtration Rates of *Daphnia* and *Simocephalus* (Crustacea: Cladocera)

**DOI:** 10.3390/plants13111504

**Published:** 2024-05-30

**Authors:** Cristian A. Espinosa-Rodríguez, Alfonso Lugo-Vázquez, Luz J. Montes-Campos, Ivan M. Saavedra-Martínez, Ma. del Rosario Sánchez-Rodríguez, Laura Peralta-Soriano, Ligia Rivera-De la Parra

**Affiliations:** 1 Grupo de Investigación en Limnología Tropical, UIICSE, FES Iztacala, Universidad Nacional Autónoma de México, Av. De los Barrios 1, Col. Los Reyes Iztacala, Tlalnepantla CP 54090, Estado de México, Mexico; caer_atl@iztacala.unam.mx (C.A.E.-R.); lugov@unam.mx (A.L.-V.); luzjazmin58@gmail.com (L.J.M.-C.); ivansaavedrarobot@gmail.com (I.M.S.-M.); rosarios@unam.mx (M.d.R.S.-R.); sorial@unam.mx (L.P.-S.); 2 Laboratorio de Fisiología Vegetal, L-204, FES Iztacala, Universidad Nacional Autónoma de México, Av. De los Barrios 1, Col. Los Reyes Iztacala, Tlalnepantla CP 54090, Estado de México, Mexico

**Keywords:** exudates, feeding rates, life table, population growth rate, zooplankton

## Abstract

Macrophytes and cladocerans represent the main antagonistic groups that regulate phytoplankton biomass; however, the mechanism behind this interaction is unclear. In laboratory conditions, we separately evaluated the effects of three submerged macrophytes (*Ceratophyllum demersum*, *Myriophyllum aquaticum*, and *Stuckenia pectinata*), as well as their exudates, and plant-associated microbiota (POM < 25 µm) + exudates on the population growth of *Daphnia* cf. *pulex* and *Simocephalus* cf. *mixtus*. Living *Ceratophyllum*, exudates, and POM < 25 µm + exudates exhibited the most robust positive effects on *Simocephalus* density and the rate of population increase (*r*). Subsequently, we examined the effects of *Ceratophyllum* on the filtration and feeding rates of *Simocephalus* and *Daphnia,* revealing significant (*p* < 0.001) promotion of filtration and feeding in *Simocephalus* but not in *Daphnia*. To elucidate the specific effects of this macrophyte on *Simocephalus* demography, we assessed selected life table variables across the same treatments. The treatments involving exudates and living *Ceratophyllum* resulted in approximately 40% longer survivorship and significantly (*p* < 0.01) enhanced fecundity. Our findings indicate that exudates from submerged macrophytes positively influence *Simocephalus* demography by increasing filtration rates, survivorship, and fecundity. This synergy suggests a substantial impact on phytoplankton abundance.

## 1. Introduction

During recent decades, a significant increase in urbanization, agriculture, livestock, deforestation, and wastewater discharges has been observed. These activities have expedited the eutrophication process in numerous water bodies around the world, leading to substantial alterations in biological communities and ecosystem dynamics [1]. Due to this environmental deterioration and global warming, eutrophication is one of the main problems facing marine and freshwater ecosystems [2]. This phenomenon drives an increase in phytoplankton biomass with associated toxic potential and reduces aquatic diversity, diminishing ecosystem services’ availability [3,4]. For this reason, several methods have been proposed to mitigate this problem. These include physical methods such as algae harvesting, dredging, water diversion, shading of lake areas, and the use of ultrasonic waves to disrupt algae cells. Chemical methods include the application of substances that kills algae, flocculants, and growth regulators [5,6,7]. However, many of these are ineffective, have harmful side effects, or are too expensive to implement [8]. In this sense, biomanipulation is a restoration method mainly applied in lakes and reservoirs, allowing for improving water quality in short periods at relatively low costs [3,8].

Submerged macrophytes have been widely used in ecological restoration projects since they are a key element in shaping aquatic communities [3,9,10]. They establish antagonistic interactions with phytoplankton by enhancing sedimentation rates, engaging in competition for light and nutrients, providing refuge for herbivores [11], and producing allelopathic compounds that reduce phytoplankton abundance [12]. Several works have shown that the allelopathic potential is more remarkable in certain species of submerged macrophytes such as *Myriophyllum* spp., *Chara* spp., *Potamogeton* spp., and *Ceratophyllum demersum* [13], with different kinds of identified compounds as major chemical mediators of this phenomenon [12,13]. These substances are released and diffused as dissolved organic carbon (DOC) into the environment, rapidly degraded by heterotrophic bacteria and thereby stimulating the microbial food web [14]. However, there is not much information at an experimental level about how these processes occur and their effect on higher trophic levels, as is the case of primary consumers such as cladocerans [15].

This interaction between macrophytes and cladocerans is relevant in biomanipulation projects since macrophytes also help to reduce phytoplankton biomass by providing shelter for zooplankton against predation, which increases herbivory rates [9]. However, the outcome of the macrophyte–cladoceran interaction is unclear since different responses have been reported in cladocerans at a demographic, physiological, and behavioral level [16,17,18,19]. Furthermore, most of these studies have been carried out in temperate systems, and several works have shown that ecological interactions in tropical and subtropical systems may differ from those observed in temperate systems [20]. Burns and Dodds [16] documented contrasting impacts of *Nitella hookeri* exudates on the filtration rates of *Daphnia carinata*, revealing both positive and negative effects depending on seasonal variations. Burks et al. [17] found that *Elodea canadensis* exudates caused a delay in maturation time and decreased egg production in *Daphnia magna*. Later, Cerbin et al. [18] mentioned that *Myriophyllum verticillatum* induced a reduction in first reproduction size and clutch size in *D. magna*, although no discernible adverse effects were observed from its exudates. Repellence effects from cladocerans towards macrophytes have also been documented [19,21]. Conversely, Espinosa-Rodríguez et al. [22] observed an enhancement in the longevity and fecundity of three *Simocephalus* species following exposure to exudates from *Egeria densa*. Montiel-Martínez [23] indicated that exudates from the floating macrophyte *Eichhornia crassipes* influenced behavior and caused positive effects on demographic parameters of littoral cladocerans, specifically *Simocephalus vetulus* and *Chydorus brevilabris*.

According to this, macrophytes may exert negative effects on pelagic cladocerans such as *Daphnia,* whereas littoral cladocerans that coevolve with macrophytes, such as those belonging to the *Simocephalus* genus, may experience positive effects. However, empirical evidence supporting these hypotheses is lacking. Consequently, the impact of submerged macrophytes, their exudates, and associated microbiota on the demographic characteristics and filtration rates of littoral and pelagic cladocerans remains uncertain. The hypothesized mechanism that explains the negative effect of macrophytes on cladocerans is associated with the release of allelopathic substances [9]; nevertheless, the chemical composition of these substances varies among macrophyte species, and their effects depend on the recipient species’ sensitivity [12,13]. On the other hand, the hypothesis suggesting a positive impact of macrophyte exudates on cladocerans relies on the fact that these substances, in the form of dissolved organic carbon (DOC), are readily decomposed by heterotrophic bacteria, which utilize them as an energy source [24]. This decomposition process activates the microbial food web, which serves as a food source for zooplankton [25], so the nature and quantity of these substances will influence bacterial production, and this will depend on the macrophyte species involved and prevailing environmental conditions [14,26]. In this study, we aimed to assess (a) the effect of three submerged macrophytes (*Ceratophyllum demersum*, *Myriophyllum aquaticum*, and *Stuckenia pectinata*), as well as their exudates and associated particulate organic matter smaller than 25 µm (POM > 25 µm), on the population growth of *Daphnia* cf. *pulex* and *Simocephalus* cf. *mixtus*; (b) the influence of *C. demersum* and *S. pectinata* on the feeding and filtration rates of *D.* cf. *pulex* and *S.* cf. *mixtus*; and (c) the effects of *C. demersum* on the survivorship and fecundity of *S.* cf. *mixtus*.

## 2. Materials and Methods

### 2.1. Test Organisms

The plankton species used for the tests (*Chlamydomonas* sp., *Daphnia* cf. *pulex*, and *Simocephalus* cf. *mixtus*) were isolated from the urban Lake Mexcalpique within Cantera Oriente. For *Chlamydomonas* sp., we directly collected the sample from the surface layer using a 50 mL Falcon tube; cladocerans were qualitatively sampled from the same layer with a zooplankton net of 64 µm mesh size. La Cantera Oriente is a place situated in the protected natural area known as “Reserva del Pedregal de San Ángel” (REPSA), Mexico City, with the coordinates ranging between 19°18′47″ and 19°19′15″ N and between 99°10′17″ and 99°10′22″ W. This region encompasses a spring and 4 shallow lakes exhibiting distinct trophic levels; therefore, it could be used as a model for assessing lake restoration strategies [27]. In these lakes, *Chlamydomonas* spp. blooms have been recorded during the cold season (November to March), while the chosen cladocerans represent the largest filter-feeding species present in these lakes. The selected aquatic plant species are distributed across several water bodies in central Mexico. *Myriophyllum aquaticum* and *Stuckenia pectinata* were isolated from the Salazar reservoir in the State of Mexico, whereas *Ceratophyllum demersum* was isolated from the channels of Xochimilco in Mexico City.

### 2.2. Culture and Maintenance of Organisms

*Chlamydomonas* sp. was isolated in 1.5% bacteriological agar Petri dishes with Bold basal medium as per the method described by Andersen et al. [28]. It was then transferred to a liquid Bold basal medium for scaling up and cultured in 500 mL transparent glass bottles at a controlled temperature of 21 ± 2 °C under aeration and constant, diffuse fluorescent light conditions. Following an incubation period of 8 days, the algae were harvested, centrifuged at 3000 RPM for 5 minutes, and resuspended in EPA medium [29]. Algal concentrations were determined using a hematocytometer. Cladoceran cultures were initiated from parthenogenetic females and maintained for 8 months in EPA medium prepared with 96 mg of NaHCO_3_, 60 mg of CaSO_4_, 60 mg of MgSO_4_, and 4 mg of KCl in 1 L of deionized water [29], at a temperature of 21 ± 2 °C under a 12:12 photoperiod, with *Chlamydomonas* sp. (at approximately 1 × 10^6^ cells mL^−1^) provided as the food source. The culture medium was replaced twice weekly. Macrophytes were collected from the field and underwent a week-long quarantine period with daily dechlorinated tap water changes to minimize associated biota. Following quarantine, they were placed in a 10% Bold basal medium under aeration, at the same temperature and photoperiod as the other organisms. To prepare the exudates and particulate organic matter less than 25 µm (POM < 25 µm), each macrophyte was rinsed with EPA medium and placed in a 5 L aquarium at a density of 1.2 g dry weight L^−1^ in 4 L of EPA medium + 10% Bold basal medium 48 h before the start of the experiment. Aquarium conditions, including light, photoperiod, and aeration, were maintained identical to those for the algae to ensure an oxygenic environment and prevent gradients. Water levels were replenished daily with EPA medium + 10% Bold basal medium.

### 2.3. Experimental Design

Four treatments were established for each macrophyte and they were evaluated separately for each cladoceran species. The treatments were (1) macrophyte exudates, (2) macrophyte exudates + associated microbiota and detritus less than 25 µm (POM < 25 µm), (3) living macrophytes at a density of 1.2 g dry weight L^−1^, and (4) control. All treatments had basal Bold medium 10% served as the nutrient source. Each treatment was replicated four times, resulting in a total of 96 experimental units (2 cladocerans × 3 macrophytes × 4 treatments × 4 replicates). For the treatments, each macrophyte was maintained as described previously. Exudates were collected by filtering the conditioned medium from each plant through 0.45 µm nitrocellulose filters. For POM < 25 µm, water containing exudates from each macrophyte was filtered through a sieve with a mesh opening size of 25 µm. In the control treatment, only EPA medium + basal Bold medium 10% was utilized.

### 2.4. Population Growth

Population growth experiments were conducted over 23–24 days. Each experimental unit comprised 100 mL transparent containers with 50 mL of medium with a concentration of 0.5 × 10^6^ cells mL^−1^ of *Chlamydomonas* sp. as the primary food source. Within each container, 10 parthenogenetic females (0.2 ind. mL^−1^) from each cladoceran species were introduced, considering cohorts consisting of 5 neonates, 3 juveniles, and 2 adults. Once the experiments began, the daily population abundance of surviving organisms was quantified, and they were subsequently transferred using a Pasteur pipette to another container corresponding to their treatment group. Based on these abundance data, population growth graphs were generated, and the daily population growth rate (*r*) was calculated using the Krebs exponential equation [30]:

*r* = (ln Nt − ln No)/t

where *r* represents the population increase rate per day, No denotes the initial population density, Nt is the final population density, and t represents the time in days. The population growth rate was calculated considering the time from the beginning of the experiment until the first point of maximum abundance in the curves for all tests. Following this calculation, growth rates were compared between treatments using a one-way ANOVA, and a post hoc Tukey test was employed using Sigmaplot 14.0 (Systat Software, Erkrath, Germany) to identify significant differences when they were observed [31].

### 2.5. Filtration and Feeding Rates

From the population growth experiments, we selected *C. demersum* and *S. pectinata* because these macrophytes had a more significant impact on the population growth curves of cladocerans. To assess the impact of *Ceratophyllum demersum* and *Stuckenia pectinata* exudates on the filtration and feeding rates of two cladoceran species, we established two transparent containers of 2 L filled with 1.5 liters of EPA medium at a controlled temperature of 21 ± 2 °C under a 12:12 photoperiod, with *Chlamydomonas* sp. provided as the food source, consistent with regular culture maintenance practices. *Ceratophyllum demersum* was added to one container at a density of 1.2 g dry weight L^−1^, while the other container received *Stuckenia pectinata* at the same density. Each container was populated with a separate cohort of either *Simocephalus* cf. *mixtus* or *Daphnia* cf. *pulex*, introduced 72 h before the experiments for acclimatization. Controls were maintained under identical conditions in two separate containers for each cladoceran species but without any plants, resulting in a total of six containers.

Prior to experimentation, cladocerans were starved for 30 min, after which five adults of *D*. cf. *pulex* (2371 ± 155 µm) and *S*. cf. *mixtus* (1781 ± 163 µm) were selected for each experimental unit, represented by transparent containers with 50 mL of the respective treatment medium with a concentration of 0.5 × 10^6^ cells mL^−1^ of *Chlamydomonas* sp. In total, 40 containers were set up (2 macrophytes × 2 cladocerans × 2 treatments × 5 replicates) with an additional 5 control containers only with *Chlamydomonas* sp. cells.

After 30 min of feeding, all experimental units were fixed with Lugol’s solution 1%, and the remaining *Chlamydomonas* sp. cells were counted using a hemocytometer in a microscope Axiostar (Carl Zeiss, Jena, Germany). Filtration rates (F) and feeding rates (f) were calculated according to Rigler [32] as follows:

F = (lnC0 − lnCt) ∗ W/t ∗ N

where C0 is the initial cell density, Ct is the final cell density, W is the medium volume in milliliters, t is the feeding time in min, and N is the number of individuals per recipient.

(f) = V(C0 − Ct)/t − N

where V is the medium volume, C0 is the initial cell density, Ct is the final cell density, t is the time, and N is the number of individuals per recipient.

Statistical comparisons were conducted independently for each cladoceran species in the presence and absence of *C. demersum* and *S. pectinata*. Thus, the results were statistically compared using one-way ANOVA and a post hoc Tukey test with Sigmaplot 14.0 (Systat Software, Erkrath, Germany) when differences were registered [31].

### 2.6. Life Table

For a detailed analysis of the observed effects on survivorship and fecundity registered in the population growth experiments and filtration and feeding rates, we conducted life table experiments using the same treatments outlined previously, focusing only on *Ceratophyllum demersum* and *Simocephalus* cf. *mixtus* given the pronounced effect of macrophytes on cladocerans registered in this combination. In each experimental unit, 10 neonates younger than 24 h were introduced each day; the number of survivors was quantified, with neonates being removed from the original cohort each day and transferred to another container corresponding to their treatment group. Life table parameters including survival (*l_x_*) and fecundity (*m_x_*) were used to calculate average lifespan (ALS), life expectancy (LE), gross (GRR) and net reproductive rate (NRR), generation time (GT), and the rate of population increase (RPI) per day following the methodology described by Krebs [30]:

Average lifespan:

$$\sum_{0}^{\infty }l_{x}$$

Life expectancy:

$$e_{0}=\frac{T_{x}}{n_{x}},T_{x}=\sum_{0}^{\infty }L_{x},L_{x}=\frac{n_{x}+n_{x+1}}{2}$$

Gross reproductive rate:

$$\sum_{0}^{\infty }m_{x}$$

Net reproductive rate:

$$R_{0}=\sum_{0}^{\infty }l_{x}m_{x}$$

Generation time:

$$T=\frac{\sum l_{x}m_{x}*x}{R_{0}}$$

Population growth rate (*r*):

$$\sum_{x=0}^{\infty }e^{-rx}l_{x}m_{x}=1$$

where *T_x_* is the number of individuals per day, *n_x_* is the number of living organisms at the beginning, *l_x_* is the probability of an organism surviving at an age class (x), *m_x_* is the fecundity at a specific age, *R*_0_ is the average number of neonates per female, and r is the rate of population increase. Differences between treatments were compared using one-way ANOVA, followed by a post hoc Tukey test with Sigmaplot 14.0 (Systat Software, Erkrath, Germany) to detect significant differences when they were registered [31].

## 3. Results

### 3.1. Population Growth

**Figure 1:**
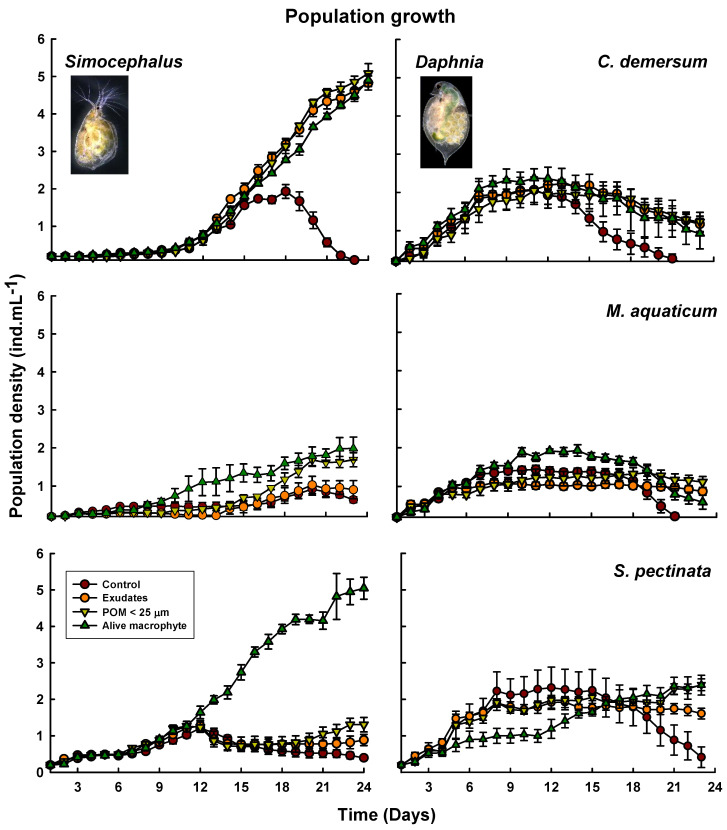
Population growth curves of *Simocephalus* cf. *mixtus* and *Daphnia* cf. *pulex* under the influence of exudates, particulate organic matter smaller than 25 µm (POM < 25 µm) + exudates, and living macrophytes derived from *Ceratophyllum demersum*, *Myriophyllum aquaticum,* and *Stuckenia pectinata*. Mean ± SE data are shown based on four replicates.

The population growth curves (Figure 1) showed that treatments incorporating exudates, POM, and living macrophytes of *Ceratophyllum demersum* resulted in an increase in *Simocephalus* abundance by close to 5 ind. mL^−1^, compared to control groups that reached densities close to 2 ind. mL^−1^. Conversely, for *Daphnia*, the presence of living *C. demersum* led to slightly higher abundances (2.37 ± 0.24 ind. mL^−1^) compared to other treatments (POM = 2.04 ± 0.35 and exudates = 2.22 ± 0.11 ind. mL^−1^) and the control (2.07 ± 0.33 ind. mL^−1^); however, the control group’s abundance declined earlier. Similarly, experiments with *M. aquaticum* also showed higher abundances in treatments for living macrophytes for both *Simocephalus* (1.98 ± 0.29 ind. mL^−1^) and *Daphnia* (1.93 ± 0.14 ind. mL^−1^) compared to other treatments, although the differences among treatments and densities were less pronounced. When *Simocephalus* was exposed to living *Stuckenia*, its population density reached 5.28 ± 0.45 ind. mL^−1^, significantly higher than the control (1.21 ± 0.09 ind. mL^−1^), while treatments with exudates and POM had slightly higher densities than the control, with 1.27 ± 0.14 ind. mL^−1^ and 1.52 ± 0.55 ind. mL^−1^, respectively. In contrast, for *Daphnia*, no clear differences were observed in the abundance among all treatments, ranging between 1.92 and 2.4 ind. mL^−1^; nevertheless, the treatment with living macrophytes showed a longer lag phase.

**Figure 2:**
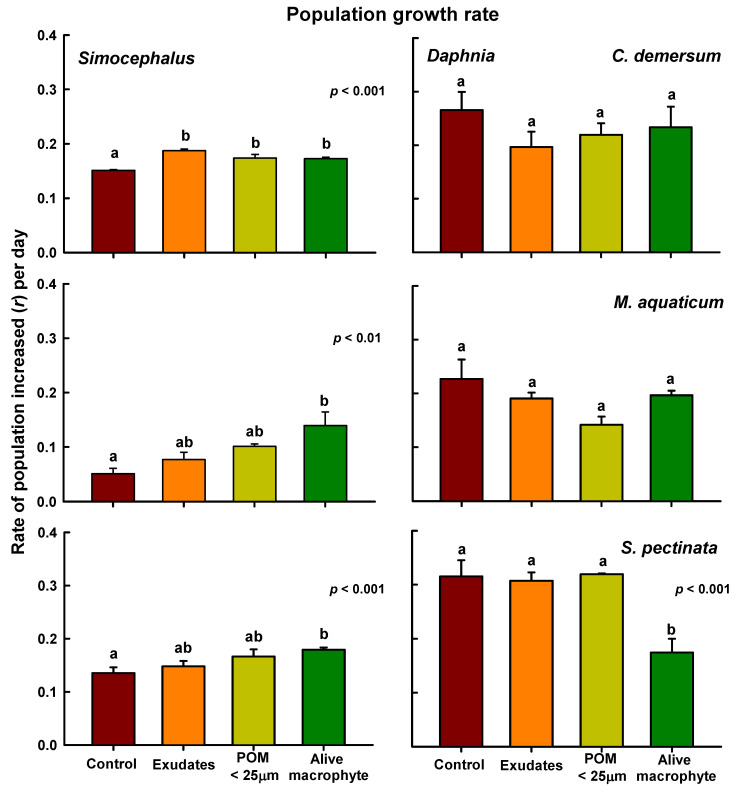
Population growth rates (*r*) per day of *Simocephalus* cf. *mixtus* and *Daphnia* cf. *pulex* under the influence of exudates, particulate organic matter smaller than 25 µm (POM < 25 µm) + exudates, and living macrophytes derived from *Ceratophyllum demersum*, *Myriophyllum aquaticum*, and *Stuckenia pectinata*. Mean ± SE data are shown based on four replicates. Bars with equal letters are not statistically significant (*p* > 0.05, Tukey test).

Overall, the *r* values ranged from 0.05 to 0.18 for *Simocephalus* and from 0.14 to 0.31 for *Daphnia,* as depicted in Figure 2. Regarding *Simocephalus*, all treatments involving exudates, POM, and living macrophytes yielded higher *r* values. However, for *Daphnia,* lower *r* values were generally observed in treatments with macrophytes or their derivates. In the ANOVA tests conducted with *Ceratophyllum* and *Simocephalus*, statistically significant differences (*p* < 0.001) were noted between the control group and the other treatments, with the highest *r* values for this cladoceran observed in the treatment involving exudates. Conversely, for *Daphnia,* no statistical differences (*p* > 0.05) were observed among the treatments. When using *M. aquaticum*, the lowest *r* values were observed for both cladocerans; however, only *Simocephalus* exhibited statistical differences (*p* < 0.01) between the control group and treatments with alive macrophytes. For *Daphnia*, no significant differences *(p >* 0.05) were detected. In the experiments with *Stuckenia*, statistical differences (*p* < 0.001) were observed between the control group and treatments with living macrophytes for *Simocephalus*, while for *Daphnia,* living *Stuckenia* significantly reduced (*p* < 0.001) its rate of population increase.

### 3.2. Filtration and Feeding Rates

The filtration and feeding rate values for *Simocephalus* significantly increased (*p* < 0.001) in the presence of *C. demersum* and *S. pectinata*, while *Daphnia* did not show statistical differences (*p* > 0.05; Table 1).

### 3.3. Life Table

**Figure 3:**
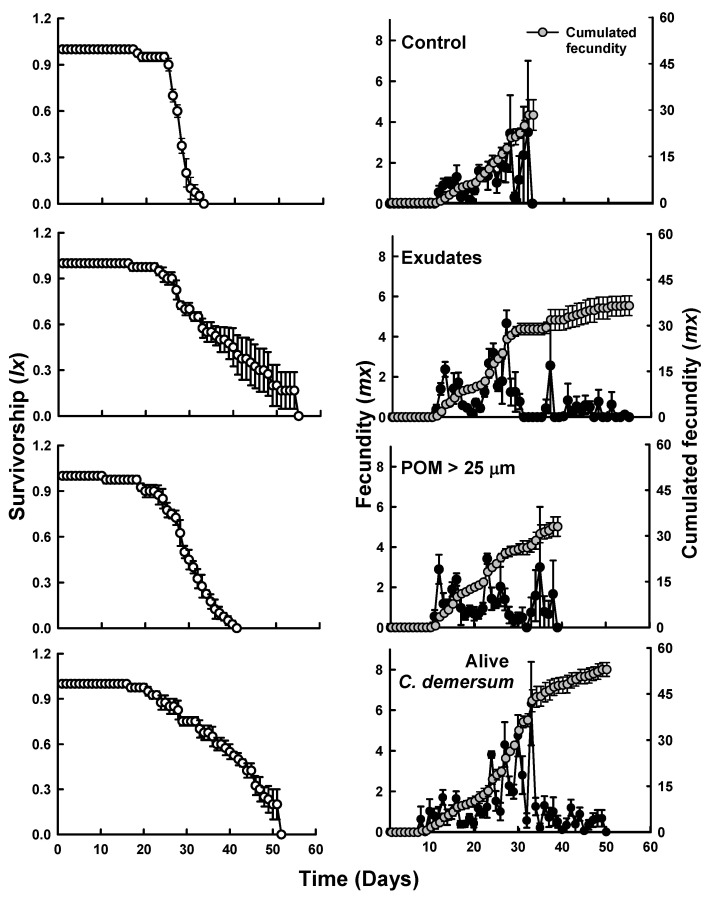
Survivorship (**left**) and fecundity (**right**) of *Simocephalus* cf. *mixtus* under the influence of exudates, particulate organic matter smaller than 25 µm (POM < 25 µm) + exudates, and living *Ceratophyllum demersum*. Mean ± SE data are shown based on four replicates.

Life table experiments showed that the survivorship (*l_x_*) of *Simocephalus* notably increased in the presence of exudates and living *C. demersum*, with individuals living up to 54 and 52 days, respectively, compared to 38 days in the POM < 25 µm treatment, and 33 days in the control group. The maximum fecundity (*m_x_*) values were relatively similar between the control and POM treatments, averaging 3.5 and 3.4 neonates per female^−1^, respectively, and cumulated fecundity around 30 neonates per female^−1^. In contrast, treatments with exudates and living macrophytes exhibited higher values of 4.6 and 6.33 neonates per female^−1^ as well as a cumulated fecundity of 36 and 52 neonates per female^−1^, respectively (Figure 3).

Table 2 displays selected life table variables for *Simocephalus*, comparing exudates, POM, and alive *C. demersum*. The average life span was similar (*p* > 0.05) between control and POM treatments, while exudates and alive *C. demersum* showed statistically different values (*p* < 0.01 and *p* < 0.001 respectively) compared to the control group. Both gross and net reproductive rates exhibited a similar trend, with similar lower values in the control group compared to the other treatments; however, exudates and POM showed no significant difference (*p* > 0.05). Notably, alive *C. demersum* resulted in statistically higher values (*p* < 0.001) compared to the other treatments. Furthermore, generation time was significantly higher for alive *C. demersum* (*p* < 0.01) and lower for POM; although, the latter was similar to the control group (*p* > 0.05). Concerning the rate of population increase, treatments with exudates and alive *Ceratophyllum* were significantly higher (*p* < 0.01 and *p* < 0.05 respectively) compared to the control group, while POM showed no statistical differences (*p* > 0.05).

## 4. Discussion

Population growth experiments with cladocerans have shown that submerged macrophytes indeed increased *Simocephalus* abundance as hypothesized, while the abundance of *Daphnia* was only marginally affected. Previous studies have presented varied responses of cladocerans to submerged macrophytes; however, the majority of research has focused on life table variables and the behavior of pelagic *Daphnia* [9,16,17,18,21], with limited attention given to *Simocephalus* and population growth responses [22,23,33].

The chemical properties of exudates derived from different macrophytes can vary significantly, leading to diverse effects on organisms [34]. For instance, *Myriophyllum spicatum* exhibits strong negative effects on cyanobacteria and green algae species through the exudation of allelopathic compounds like tellimangrandin II and ellagic acid. Similarly, *Ceratophyllum demersum* releases allelopathic sulfur compounds targeting diatoms [12,13]. In a meta-analysis examining the inhibition mechanisms of submerged macrophytes on algae, Liu et al. [35] found that the inhibition of algae by submerged plants is species-specific, with *Ceratophyllum demersum* demonstrating the strongest allelopathic effect on algae.

When assessing the impact of macrophytes on cladocerans, Gutierrez and Mayora [15] emphasized variations in phenols and chromophoric dissolved organic matter (DOM) exudates among different macrophyte species, resulting in changes in the avoidance behavior of cladocerans and copepods. Additionally, various species of zooplankton exhibited divergent responses to the same species of macrophyte. Moreover, additional research indicates that the association between macrophytes and cladocerans varies depending on the particular species involved [15,36]. Therefore, the effect of macrophyte exudates relies on both the specific macrophyte species and the susceptibility of plankton.

Most research on macrophyte–*Daphnia* interactions has revealed negative impacts of macrophytes on *Daphnia*, attributed to physical and chemical factors such as accelerated sedimentation of food particles due to macrophyte structure, macrophytes acting as barriers to swimming [18], and *Daphnia* avoiding macrophytes due to heightened predation risk from fish [15,19,21]. Additionally, numerous studies indicate that allelopathic substances exuded by macrophytes can reduce fecundity, delay maturation, and diminish *Daphnia* growth [9]. However, the effects of DOM exudated from macrophytes on cladocerans have been poorly assessed in laboratory conditions [15].

Burks et al. [17] found that *Daphnia* exposed to chemical exudates from macrophytes experienced delayed maturation and reduced fecundity, implying indirect costs to *Daphnia* due to the allelopathic inhibition of their algal food source. However, in our investigation, distinct negative impacts on the cladoceran population were not evident; instead, we observed a prolonged lag phase in the growth population of *Daphnia* exposed to *Stuckenia pectinata*; consequently, the growth rate was diminished, even though the abundances across all treatments remained similar.

On the other hand, the advantageous impacts of macrophyte exudates on *Simocephalus* spp. were evidenced by the enhancement of lifespan, survivorship, and fecundity through allelochemicals released by *Egeria densa* [22]. These findings align with our own, revealing a positive influence of submerged macrophyte exudates on *Simocephalus* demography and filtration rates. Notably, age-specific survivorship showed a 40% increase, accompanied by elevated average lifespan and reproductive rates in treatments involving exudates and alive plants. Additionally, positive effects on the population density of *Macrothrix triserialis*, *Diaphanosoma birgei*, *Simocephalus mixtus*, and *Daphnia mendotae* from *Egeria densa*’s allelochemicals were reported as well [33].

Despite these advancements, our understanding of the effects of macrophyte exudates and secondary metabolites on aquatic herbivores remains limited [37]. Some explanations attribute these outcomes to hormesis, occurring under low-stress conditions where reduced toxicity promotes reproduction, longevity, and survival [38]. These conditions are often found in littoral vegetated areas, potentially explaining the high zooplankton abundances observed there. Moreover, allelochemicals released by macrophytes have both negative and positive effects on different species of algae [39], suggesting a similar dual impact on zooplankton.

From an eco-evolutionary perspective, *Simocephalus* has coevolved in the littoral with presence of macrophytes [40], where allelopathic exudates are prevalent. Consequently, this has shaped the conditions of its habitat, leading to lower fitness in the absence of such conditions, as observed in the control group. While the survivorship and fecundity of *Simocephalus* increased in the presence of exudates and live *Ceratophyllum*, a longer generation time indicated a trade-off between these variables. The presence of phenolic compounds and tannins negatively affected herbivores’ feeding rates, yet some secondary metabolites, including tannins, exhibited beneficial effects against pathogens and stressors, akin to their roles in terrestrial herbivores [37]. Additionally, phenolic compounds are recognized as antioxidant compounds [41], and recent studies have identified anticancer phenolic compounds in *C. demersum* [42], underscoring macrophytes as a rich source of beneficial substances. However, our knowledge regarding the benefits of secondary metabolites exuded from macrophytes in aquatic systems remains sparse [37]. Thus, identifying and studying these beneficial secondary metabolites in macrophyte exudates presents a critical opportunity to understand processes in freshwater ecology.

Macrophyte exudates constitute a substantial portion of total dissolved organic matter (DOM) in vegetated habitats [15], and numerous studies elucidate their role in enhancing primary and secondary productivity [43]. Mesocosm experiments conducted by Balzer et al. [44] delineate how allochthonous DOM stimulates autotrophs and mixotrophs, playing a pivotal role in zooplanktonic secondary production. The DOM generated by submerged macrophytes primarily comprises protein-like substances exhibiting high activity, with discernible variations observed among different aquatic plants. Some investigations have documented that DOM significantly influences alterations in microbial community composition [43]. In our population growth experiments, the treatments involving particulate organic matter smaller than 25 µm (POM < 25 µm) were statistically comparable to exudates concerning *Simocephalus* and *Daphnia*. This suggests that the microbiota and detritus associated with macrophytes may not serve as a substantial nutritional food source for cladocerans under these conditions.

Feeding behavior in daphnids is characterized by a generalist approach, wherein the largest ingestible particle size is determined by the frontal opening of the carapace, while the smallest particles are limited by the mesh size of the filtration setules in the thoracopods [45]. Mesh sizes vary among different cladoceran species, ranging from 0.16 µm for *Diaphanosoma* to 4.7 µm in *Sida*. For *Daphnia* species, mesh size ranges from 0.23 to 0.45 µm for *D. cucullata* up to 0.56 to 1.8 µm for *D. hyalina* [46]; however, data for *Simocephalus mixtus* are currently unavailable. Despite their ability to ingest a wide range of particle sizes, the optimal food size range for cladocerans typically spans from 3 to 20 µm [45]. Larger cladocerans such as *D. pulex* exhibit up to threefold higher filtration efficiency on 20 µm algal particles compared to bacteria, which are usually smaller than 2 µm, thereby safeguarding picoplankton from extensive grazing by large cladocerans [45,47]. Consequently, the bacteria associated with submerged macrophytes and produced through exudated dissolved organic matter (DOM) may not be efficiently consumed by the relatively large *Simocephalus* cf. *mixtus* (1781 ± 163 µm) and *Daphnia* cf. *pulex* (2371 ± 155 µm) utilized in our experiments.

Detritus represents another crucial component of POM. For littoral *Simocephalus*, the fatty acid composition aligns with that of littoral particulate matter, underscoring its relevance as a resource for littoral species. In contrast, *Daphnia* primarily derives nutrients from phytoplankton [48]. Cladocerans typically recycle DOM through the microbial loop, wherein they readily consume flagellates and certain ciliates that prey on bacteria [46]. However, our observations in the aquarium containing *C. demersum* revealed predominantly amoebas and hypotrich ciliates among the protozoans, which are less susceptible to consumption by filtering cladocerans due to their surface association [49].

In terms of filtration and consumption rates, *D.* cf. *pulex* demonstrated higher cell consumption and water filtration compared to *S.* cf. *mixtus* in control conditions, consistent with findings from previous research [50]. This discrepancy can be attributed to the larger size of *Daphnia* relative to *Simocephalus* [51]. The filtration rates of cladocerans are influenced by factors such as food quality and quantity, particle size, and the size of the cladoceran itself [48]. Therefore, we anticipated higher filtration rates in *Daphnia* compared to *Simocephalus*, as reflected in our control data. However, *Simocephalus* exposed to exudates from *Ceratophyllum* and *Stuckenia* showed a statistically significant increase in filtration rates, approaching the maximum reported values for its size [51]. In contrast, the filtration rates of *Daphnia* were not statistically affected by macrophyte exudates and remained consistent with previous reports [52].

Despite the crucial role of filter feeders and the filtration process in maintaining water clarity [53], there is limited information available regarding the effects of macrophyte exudates on cladoceran filtration and feeding rates. Typically, the filtration rates of cladocerans range from 0.9 to 135 mL per individual over a 24-h period [48], which aligns with our data for both *Daphnia* and *Simocephalus*. In summary, the presence of macrophytes and their exudates can influence the filtration and consumption rates of *Simocephalus*, but not *Daphnia*. This has significant implications for managing algal blooms and preserving water quality in aquatic ecosystems.

## 5. Conclusions

In this study, we investigated the effects of three submerged macrophytes, their exudates, and associated particulate organic matter on the population growth and filtration rates of *Simocephalus* and *Daphnia*, highlighting the complexity of interactions between aquatic plants and zooplankton. Our results reveal significant differences in the responses of different cladoceran species to macrophyte exudates, emphasizing the importance of considering species-specific responses in ecological studies and biomanipulation trials. Submerged macrophytes had positive effects on the abundance and rate of population increase of *Simocephalus* cf. *mixtus* through the exudation of chemical substances, with *Ceratophyllum demersum* showing the strongest positive effect on survivorship, average lifespan, and reproductive rates of *S.* cf. *mixtus*, with no clear negative effects on *Daphnia* cf. *pulex*. This differential response underscores the specificity of interactions between zooplankton and macrophyte exudates. The increase in filtration and feeding rates in the presence of macrophyte exudates may partially explain the positive effects registered on demographic parameters. The findings of this study have implications beyond individual species dynamics, encompassing ecological consequences. The influence of macrophyte exudates on cladoceran populations may have cascading effects on water quality and the control of algal proliferation. Understanding these complex interactions is crucial for effective ecosystem management and restoration efforts.

In summary, our study provides valuable insights into the ecological importance of macrophyte exudates in freshwater ecosystems. By elucidating the underlying mechanisms of zooplankton responses to macrophyte-derived compounds, we lay the groundwork for future research.

## Figures and Tables

**Table 1:** Filtration and feeding rates of *Simocephalus* cf. *mixtus* and *Daphnia* cf. *pulex* in the presence and absence of *Ceratophyllum demersum* and *Stuckenia pectinata*. Means ± SE are based on five replicates. Data with equal letters are not statistically significant (*p* > 0.05, Tukey test).

		*Simocephalus* cf. *mixtus*	*Daphnia* cf. *pulex*
**Filtration rates**	Control	1.01 ± 0.19 ^a^	2.14 ± 0.24 ^a^
**(mL ind. h^−1^)**	*C. demersum*	4.07 ± 0.52 ^b^	1.99 ± 0.32 ^a^
	*S. pectinata*	3.02 ± 0.30 ^c^	1.88 ± 0.15 ^a^
**Feeding rates**	Control	0.81 ± 0.15 ^a^	1.63 ± 0.17 ^a^
**×10^5^ (cells ind. h^−1^)**	*C. demersum*	2.87 ± 0.31 ^b^	1.52 ± 0.22 ^a^
	*S. pectinata*	2.22 ± 0.20 ^c^	1.45 ± 0.25 ^a^

**Table 2:** Selected life table variables of *Simocephalus* cf. *mixtus* under the influence of exudates, particulate organic matter smaller than 25 µm + exudates (POM < 25 µm), and living *Ceratophyllum demersum*. Mean ± SE data are shown based on four replicates. Data with equal letters are not statistically significant (*p* > 0.05, Tukey test). ALS = Average life span; GRR = gross reproductive rate; NRR = net reproductive rate; GT = generational time; RPI = rate of population increase.

Treatments	Variable	*Simocephalus*
**Control**	ALS (days)	26.6 ± 0.33 ^a^
**Exudates**		37.9 ± 2.45 ^b^
**POM < 25 µm**		28.7 ± 0.87 ^a^
**Alive *C. demersum***		39.0 ± 1.43 ^b^
**Control**	GRR (neonates per female^−1^)	23.3 ± 1.23 ^a^
**Exudates**		37.1 ± 2.64 ^b^
**POM < 25 µm**		38.4 ± 2.86 ^b^
**Alive *C. demersum***		52.6 ± 3.63 ^c^
**Control**	NRR (neonates per female^−1^)	18.1 ± 0.67 ^a^
**Exudates**		28.7 ± 1.81 ^b^
**POM < 25 µm**		24.5 ± 1.13 ^ab^
**Alive *C. demersum***		41.1 ± 2.63 ^c^
**Control**	GT (days)	20.6 ± 0.65 ^ab^
**Exudates**		22.3 ± 0.4 ^ac^
**POM < 25 µm**		19.2 ± 0.36 ^b^
**Alive *C. demersum***		24.6 ± 1.05 ^c^
**Control**	RPI (*r*)	0.15 ± 0.06 ^a^
**Exudates**		0.24 ± 0.02 ^b^
**POM < 25 µm**		0.18 ± 0.07 ^a^
**Alive *C. demersum***		0.22 ± 0.01 ^b^

## Data Availability

Data are available from the authors upon reasonable request.
